# Cervical spinal cord injury leads to injury and altered metabolism in the lungs

**DOI:** 10.1093/braincomms/fcad091

**Published:** 2023-03-28

**Authors:** Emily E Huffman, Brittany E Dong, Harrison A Clarke, Lyndsay E A Young, Matthew S Gentry, Derek B Allison, Ramon C Sun, Christopher M Waters, Warren J Alilain

**Affiliations:** Department of Neuroscience, University of Kentucky College of Medicine, Lexington, KY 40508, USA; Spinal Cord and Brain Injury Research Center, University of Kentucky College of Medicine, Lexington, KY 40508, USA; Department of Physiology, University of Kentucky College of Medicine, Lexington, KY 40508, USA; Department of Neuroscience, University of Kentucky College of Medicine, Lexington, KY 40508, USA; Markey Cancer Center, University of Kentucky, Lexington, KY 40508, USA; Department of Molecular and Cellular Biochemistry, University of Kentucky, Lexington, KY 40508, USA; Markey Cancer Center, University of Kentucky, Lexington, KY 40508, USA; Department of Molecular and Cellular Biochemistry, University of Kentucky, Lexington, KY 40508, USA; Markey Cancer Center, University of Kentucky, Lexington, KY 40508, USA; Department of Pathology and Laboratory Medicine, University of Kentucky College of Medicine, Lexington, KY 40508, USA; Department of Neuroscience, University of Kentucky College of Medicine, Lexington, KY 40508, USA; Spinal Cord and Brain Injury Research Center, University of Kentucky College of Medicine, Lexington, KY 40508, USA; Department of Physiology, University of Kentucky College of Medicine, Lexington, KY 40508, USA; Saha Cardiovascular Research Center, University of Kentucky College of Medicine, Lexington, KY 40508, USA; Department of Neuroscience, University of Kentucky College of Medicine, Lexington, KY 40508, USA; Spinal Cord and Brain Injury Research Center, University of Kentucky College of Medicine, Lexington, KY 40508, USA

**Keywords:** spinal cord injury, lung injury, metabolism

## Abstract

High-cervical spinal cord injury often disrupts respiratory motor pathways and disables breathing in the affected population. Moreover, cervically injured individuals are at risk for developing acute lung injury, which predicts substantial mortality rates. While the correlation between acute lung injury and spinal cord injury has been found in the clinical setting, the field lacks an animal model to interrogate the fundamental biology of this relationship. To begin to address this gap in knowledge, we performed an experimental cervical spinal cord injury *(N* = 18*)* alongside sham injury (*N* = 3) and naïve animals (*N* = 15) to assess lung injury in adult rats. We demonstrate that animals display some early signs of lung injury two weeks post-spinal cord injury. While no obvious histological signs of injury were observed, the spinal cord injured cohort displayed significant signs of metabolic dysregulation in multiple pathways that include amino acid metabolism, lipid metabolism, and N-linked glycosylation. Collectively, we establish for the first time a model of lung injury after spinal cord injury at an acute time point that can be used to monitor the progression of lung damage, as well as identify potential targets to ameliorate acute lung injury.

## Teaser

Huffman *et al.* establish for the first time a model of lung injury after spinal cord injury at an acute timepoint. While the correlation between lung injury and spinal cord injury is established in the clinical setting, the field lacks an animal model to interrogate the biology of this relationship.

## Graphical Abstract

**Graphical abstract:**
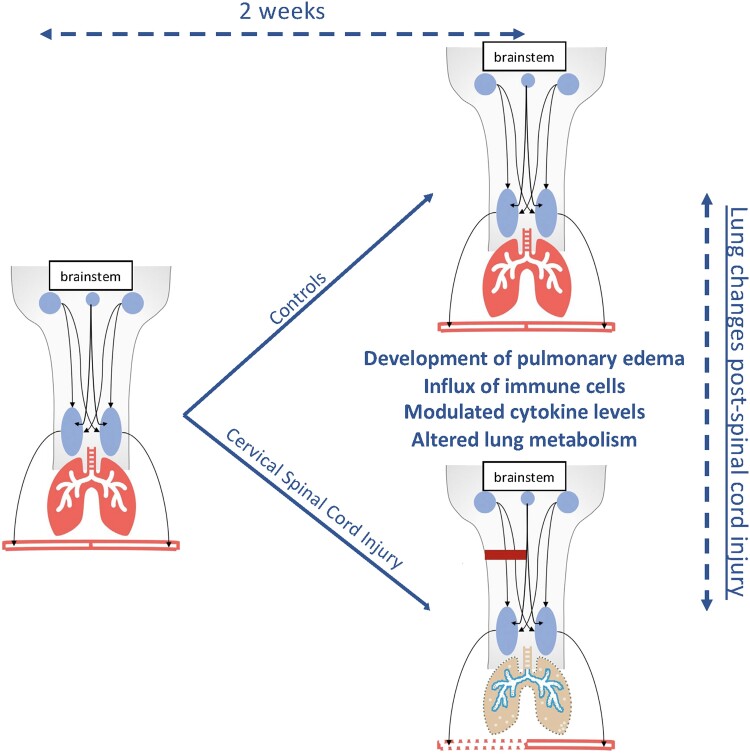

## Introduction

Cervical spinal cord injuries (SCIs) can silence descending signals from the brainstem that control diaphragmatic activity. This injury can consequently impair breathing, making individuals prone to the development of profound respiratory complication.^1-3^ In fact, respiratory complication is the leading cause of morbidity and mortality for cervical SCI individuals.^4^ Two common forms of these complications found in SCI are acute lung injury (ALI) and its most severe form, acute respiratory distress syndrome (ARDS). Both complications introduce significant risks of mortality that may occur in association with a myriad of etiologies such as trauma, pneumonia, shock, aspiration, and sepsis.^5,6^ Inflammatory responses to these neutrophil-mediated injuries contribute damage to the vascular endothelium and alveolar epithelium, where long-term repercussions can persist even after resolution of lung injury.^7,8^

Recent evidence suggests that persons with an SCI hold a significant risk of developing ARDS/ALI. Most notably, those with a cervical injury hold the greatest risk and the highest mortality rate compared to other levels of SCI.^9^ This observation is not limited to SCI alone. In fact, ARDS/ALI has been found in humans following other forms of neurotrauma, including vertebral column fracture, stroke, and traumatic brain injury.^9-11^ In addition to the implications for lung damage and inhibition of ventilatory function, there is evidence that ARDS/ALI also impacts neurologic outcomes following central nervous system injury.^12^ These substantial consequences present an exigent call to characterize the peripheral repercussions of SCI. While many comorbidities and complications of SCI have gained attention in recent years, the effect of a cervical SCI on the lungs, especially the characterization of their health, function, and cellular composition, has not been explored in an animal model.^13,14^ Currently, the field lacks an animal model to interrogate the molecular underpinning of ALI after cervical SCI, which is a major barrier to identifying therapeutic windows and treatment opportunities.

In this study, we report a model of cervical SCI-induced ALI/ARDS in rats. We identified classic markers of lung injury after SCI that are present two weeks after the initial time of cervical injury. We performed a thorough assessment of lung injury including severity of edema, protein infiltration in the bronchoalveolar lavage fluid (BAL), neutrophil and cellular levels, cytokine concentrations, metabolism, and histopathological reports; which enabled a thorough assessment of lung injury. This study demonstrates that features of lung injury and changes in metabolism ensue after experimental cervical SCI, establishing a promising model of ALI/ARDS at an acute time point that can be used to characterize and monitor the progression of lung injury after SCI, as well as provide a means to test potential therapies.

## Materials and methods

### Animals

All procedures were in accordance with Institutional Animal Care and Use Committee guidelines. Thirty female Sprague Dawley rats (12 months) from Charles River Laboratories, Inc., were housed under standard light/dark cycles with *ad libitum* access to food and water. Animals were monitored daily following surgery.

### C2 hemisection spinal cord injury

C2 hemisection (C2Hx) was conducted as previously described.^15^ Briefly, animals were anaesthetized with 4% isoflurane and prepared for surgery by shaving and cleaning from the ears to the shoulders with alternating bouts of Betadine and 70% ethanol. Body temperature was maintained throughout the procedure by a heating pad placed underneath the animal. Under aseptic conditions, a dorsal midline incision was performed to expose the cervical region of the spinal column. A C2 laminectomy allowed for visualization of the dorsal cervical spinal cord. After durotomy and laminectomy, a 27-gauge needle was bent and inserted into midline at C2 and dragged laterally to sever the left hemi-cord. This process was repeated for a total of three times to ensure completion. After C2Hx, the layers of musculature were sutured and the skin was clipped closed. The animals were given Buprenorphine (0.02–0.05 mg/kg), Carprofen (5 mg/kg), and saline when removed from anesthesia. These post-operative care procedures were continued for three days following injury. Sham injury followed the above procedure, excluding hemisection.

### Experimental design

Fifteen rats were given a cervical SCI, another fifteen were naïve. Sham animals were not initially included as the injury mimics a vertebral column fracture, which induces ALI/ARDS in the clinical setting.^9^ The SCI and naïve animals were randomly assigned to three groups: edema group, BAL group, and histology/matrix-assisted laser desorption/ionization mass spectrometry imaging (MALDI-MSI) group. These three groups each contained five naïve and five SCI animals, allowing 20 lungs to study. Following these studies, the lungs of an additional three sham injured and three SCI animals were analyzed using gas chromatography-mass spectrometry (GCMS). All individuals involved in experimentation, outcomes, and data analysis were blinded.

### Collection and sacrifice

Animals were deeply anesthetized under 4% isoflurane (SomnoSuite Low-Flow Anesthesia System) and subsequently overdosed by isoflurane administration 2 weeks after SCI. Rats received a laparotomy and the abdominal aorta was transected as a secondary means of sacrifice. All procedures were conducted immediately post-mortem.

### Bronchoalveolar lavage fluid collection

Following sacrifice, an 18-gauge tracheal cannula connected to a 3 mL syringe filled with 2 mL of sterile, cold 0.1% ethylenediaminetetraacetic acid was inserted without force into the left bronchus and ligated with suture. The syringe was compressed for 1 minute. Insertion was verified by inflation of the lung. After a 1-minute pause, BAL fluid was withdrawn for another minute and deposited into sterile vials on ice. This was repeated by attaching a second syringe filled with 2 mL of sterile solution to the inserted cannula. Keeping the cannula in the respiratory tract, the needle was then guided into the right primary bronchus and the procedure was repeated. In total, 4 mL of lavaged fluid were collected from each lung.

### Pulmonary edema

Following sacrifice, the lungs were removed *en bloc,* dissected, blotted, and desiccated in a hybridization oven at 65°C. The wet/dry weight ratio for each lung was calculated by dividing the mass of the wet lung by the mass of the dry lung.

### Histology

A 16-gauge blunted cannula was inserted into the trachea and secured with suture. Sixty-milliliter cold PBS was pushed through the right ventricle of the heart to clear the lungs of blood. The cannulated lungs were removed from the pleural cavity and pressure fixed *en bloc* (25 cm H_2_O) with 10% formalin. Lungs were dissected from each other, processed, embedded in paraffin, serially sectioned at 5 µm, and mounted on slides. Injury severity was assessed by a blinded pathologist after hematoxylin and eosin (H&E) staining.

### Cytokine assessment

Collected BAL was centrifuged at 200×g for 10 minutes at 4°C, immediately freezing the supernatant. Tumor necrosis factor alpha (TNF-α), Interleukin 1 beta (IL-1β), Interleukin 4, Interleukin 6 (IL-6), and keratinocyte chemoattractant/human growth-related oncogene (KC/GRO) concentrations were measured in the supernatant via meso scale discovery (MSD) assay, which uses electrochemiluminescence as a detection technique for higher sensitivity.

### Protein assessment

Protein concentrations were measured via bicinchoninic acid assay. Briefly, 25 µL of BAL supernatant were mixed with 100 µL of reagent and incubated at 37°C for 30 minutes. Protein concentration was analyzed at 562 nm and compared to a standard curve constructed using known protein concentrations.

### Cellular assessments

Pellets from centrifuged BAL were resuspended. Counts were performed with Trypan Blue exclusion and approximately 30 000 cells were cytospun onto microscopy slides. Cells were fixed and stained with a Diff-Quik stain. Total neutrophils were measured by morphologic identification.

### Matrix-assisted laser desorption ionization mass spectrometry imaging

Formalin-fixed paraffin embedded (FFPE) lungs were processed as previously described.^15,16^ In brief, FFPE lungs were sectioned at 5 µm on glass slides. Tissues were dewaxed and rehydrated followed by an antigen retrieval process in citraconic anhydride buffer. Recombinant PNGase F (0.1 µg/mL) was applied by a M5 TM robotic Sprayer (HTX Technologies LLC, Chapel Hill, NC). Following incubation in a humidity chamber for 2 hours, slides were desiccated for 24 hours. The following day, 7 mg/mL of alpha-Cyano-4-hydroxycinnamic acid in 50% acetonitrile with 0.1% TFA were applied to each slide by the robotic sprayer. Slides were subsequently stored in a desiccator prior to MALDI-MSI analysis.

A Waters Synapt G2Si mass spectrometer (Waters Corporation, Millford, MA) equipped with a Nd:YAG UV laser with a spot size of 100 µm was used to detect N-glycans at *X* and *Y* coordinates of 100 µm. Following data acquisition, mass spectra were analyzed by High Definition Imaging Software (Waters Corporation) for mass range 500–3500 m/z. Three regions of interest were determined for each lung; pixel intensities were averaged and normalized by total ion current. Representative N-glycans were generated by Glycworkbench.

### Gas chromatography-mass spectrometry

Lungs were snap-frozen in liquid nitrogen and put into cryostorage after surgical removal. Lungs were processed as previously described.^17-19^ Frozen lungs were transferred to a mircovial set for use with a Freezer/Mill Cryogenic Grinder (SPEX SamplePrep model 6875D). Tissue was pulverized to 10 µm particles. Metabolites were extracted from ∼20 mg of tissue from addition of 1 mL of 50% methanol. One microliter of 20 µM L-norvaline (procedural, internal control) was added. Samples were centrifuged at 21 130 rcf for 10 minutes at 4°C, separating into polar (aqueous layer) and insoluble pellet (protein/DNA/RNA/glycogen). The pellet was subsequently washed four times with 50% methanol and once with 100% methanol to remove polar contaminants. The pellet was then hydrolyzed in 200 µL of 3N hydrochloric acid and then hydrolyzed metabolites were extracted with 200 µL of 100% methanol. The polar and pellet fraction was dried at 10–3 mBar using a SpeedVac (Thermo) followed by derivatization. The insoluble pellet was then hydrolyzed.

Dried polar and insoluble samples were derivatized by the addition of 50 µL 20 mg/mL methoxyamine hydrochloride in pyridine, vortexed thoroughly, and incubated for 1.5 hours at 30°C. Sequential addition of 80 µL of N-methyl-trimethylsilyl-trifluoroacetamide was performed, followed with an incubation time of 30 minutes at 37°C with thorough vortexing between addition of solvents. The mixture was then transferred to an amber, v-shaped glass chromatography vial and analyzed by GCMS.

An Agilent 7800B gas chromatography coupled to a 5977B mass spectrometry (GCMS) detector was used for this study. A modified temperature gradient was used for GC: Initial temperature was 130°C, held for 4 minutes, rising at 6°C/minute to 243°C, rising at 60°C/minute to 280°C, held for 2 minutes. The electron ionization energy was set to 70 eV. Scan (m/z: 50–800) and full scan mode were used for metabolomics analysis. Mass spectra were translated to relative metabolite abundance using MassHunter MS quantitative software matched to the FiehnLib metabolomics library (available through Agilent) for retention time and fragmentation pattern. Relative abundance was corrected for recovery using the L-norvaline standard and adjusted to protein input represented by the sum of amino acids from the pellet fraction also analyzed by GCMS.

### Statistical analysis

All numerical data were analyzed by a two-tailed unpaired student *t*-test. For all analyses, *P* < 0.05 was considered significant. Following MALDI-MSI analysis, MetaboAnalyst 5.0 was used to generate heatmaps and principal component analyses.^20^

### Data availability

Raw data were generated at the University of Kentucky. All data will be uploaded to the Open Data Commons for Spinal Cord Injury. Derived data supporting the findings of this study are available from the corresponding author on request.

## Results

### Pulmonary edema developed after SCI

**Figure 1:**
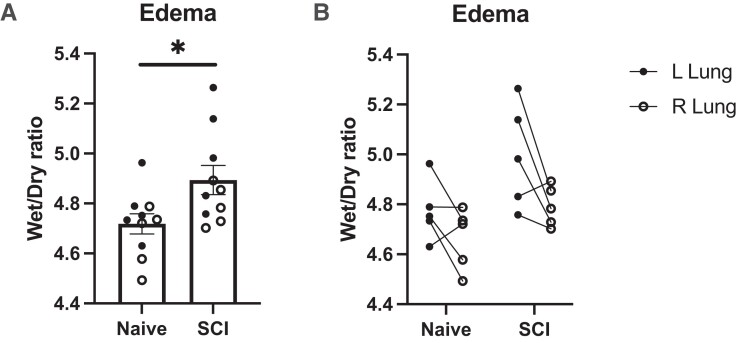
**Wet/dry ratios.** Wet/dry ratios of excised lungs were significantly increased two weeks post-SCI compared with naïve rats (**A**; *N* = 10 lungs, *P* = 0.0240). The left lung presented a trend towards a greater wet/dry ratio (**B**; *N* = 5 lungs). The significance of this finding was not observed. For all analyses, a two-tailed unpaired student *t*-test was used. *P* < 0.05 was considered significant.

To determine whether there was an excess of fluid in the lungs after acute SCI, we evaluated the wet/dry ratio from the lungs of naïve and 14 *dpi* SCI animals. The wet/dry ratio has been found to correlate with the results of other measures of lung injury.^21^ We found that C2Hx elicited a significant increase in the wet/dry ratio of the lungs of SCI animals (4.89 ± 0.06) compared to naïve lungs (4.72 ± 0.04) (*P* = 0.0240) (Fig. 1A). Wet/dry ratios were increased in the left lungs compared to the right lungs following C2Hx (*N* = 5), however this finding was not significant, potentially due to small numbers of lungs (Fig. 1B).^22^

### SCI increases BAL cell counts but not protein levels in rats

**Figure 2:**
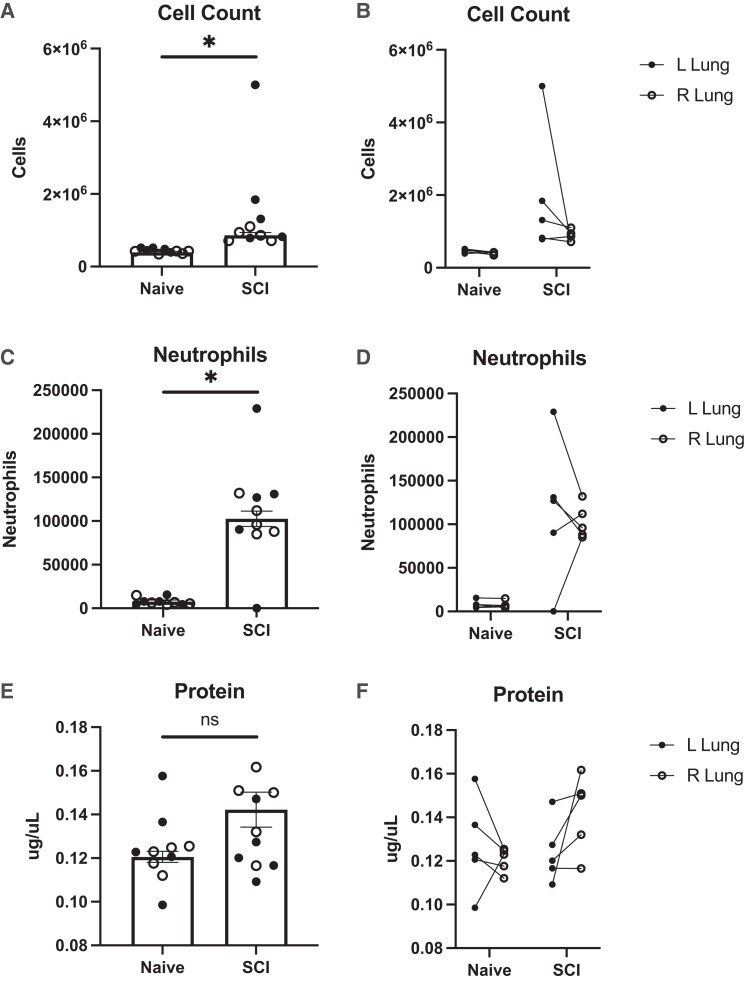
**Cellular counts.** Changes in BAL cell numbers and neutrophil counts were significantly increased two-weeks post-SCI compared with naïve rats. Measurements of total cell count (**A-B**), neutrophils (**C-D**), and protein concentration (**E-F**) in bronchoalveolar lavage fluid (BAL) from naïve and two weeks post-SCI rats. There was a significant increase in total cell count and neutrophils following SCI (A, C; *N* = 10 lungs, *P* = 0.0300; *P* = 0.0001), but no detected change in protein (E *N* = 10 lungs, *P* = 0.2324). The left lung trends towards a greater cell count and neutrophil levels following SCI (B, D; *N* = 5 lungs). However, the significance of this finding was not observed possibly due to small Ns. For all analyses, a two-tailed unpaired student *t*-test was used. *P* < 0.05 was considered significant.

To further characterize the severity of injury, we evaluated the migration of immune cells into the lungs after SCI.^23^ Following SCI, the total number of cells increased significantly (1.41 ± 0.04 ×10^7^) compared to naïve lungs (4.31 ± 0.02 ×10^6^) (*P* = 0.0300). Additionally, a differential cell count revealed that the number of neutrophils was elevated in SCI animals (1.10 ± 0.18 ×10^5^) compared to naïve (7.79 ± 1.29 ×10^3^) (*P* = 0.0001) (Fig. 2 A–D). Using these measurements, we estimated that neutrophils comprised 1.8% of the BAL prior to injury and 8.6% post-SCI. These results show that not only did the number of cells increase in the BAL, but the percentage of neutrophils found in the lungs also increased after SCI. However, as shown in Fig. 2E and F, protein levels in the BAL were not significantly increased in the SCI rats (0.133 ± 0.006 μg*/*μL) compared to naïve (0.124 ± 0.005 μg*/*μL) (*P* = 0.2324) (Fig. 2E and F). Again, the left lungs had greater cell counts and larger neutrophil counts compared to the right lung, but this finding was not significant (*N* = 5).

### SCI modulates cytokine levels in BAL

**Figure 3:**
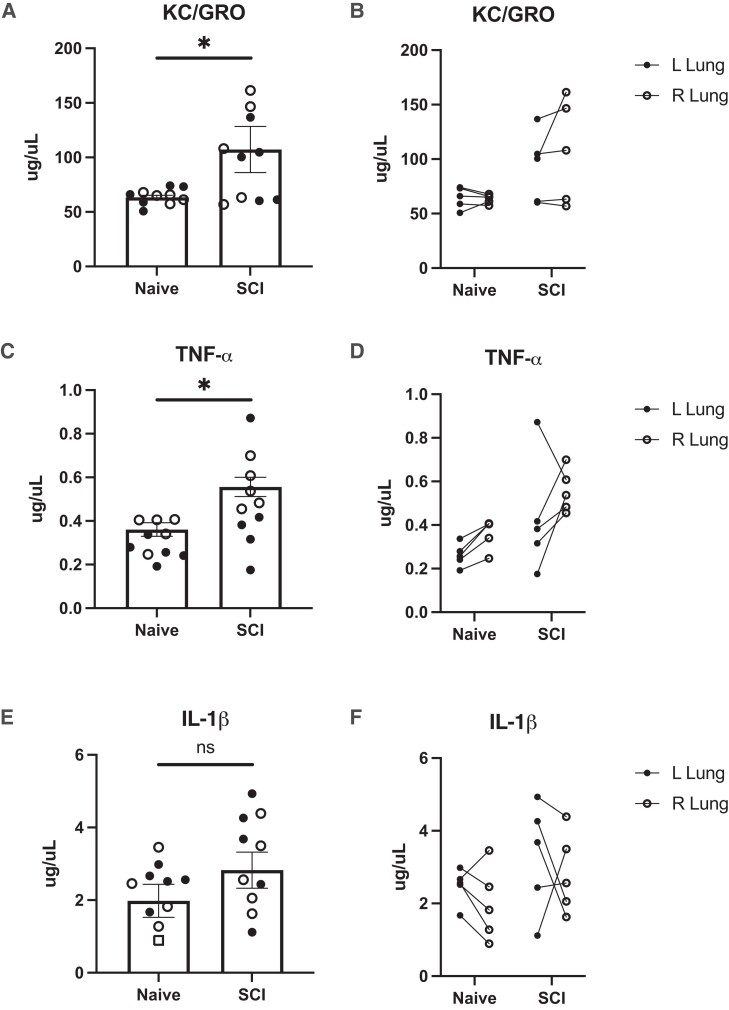
**Cytokine levels.** Levels of KC/GRO and TNF- α were significantly increased two-weeks post-SCI compared with naïve rats. Measurements of KC/GRO (**A-B**), TNF-α (**C-D**), and IL-1β (**E-F**) in bronchoalveolar lavage fluid (BAL) from naïve and two weeks-post SCI rats. There was a significant increase in levels of KC/GRO and TNF-α following SCI (A, C; *N* = 10 lungs, *P* = 0.0103; *P* = 0.0139), however, there was no significant change in levels of IL-1β following SCI (*P* = 0.1007). Side-specific differences were not found following SCI (B, D, F; *N* = 5 lungs). For all analyses, a two-tailed unpaired student *t*-test was used. *P* < 0.05 was considered significant.

To further investigate the increase in cells found in the BAL after SCI, we measured levels of the neutrophil chemoattractant KC/GRO, as well as other indicators of inflammation including the cytokines TNF-α, IL-1β, and IL-6, whose concentrations have been observed to increase following lung injury.^24-26^ MSD results showed significantly increased levels of KC/GRO (100.0 ± 12.33 μg*/*μL) and TNF-α (0.50 ± 0.06 μg*/*μL) in SCI animals compared to naïve (64.04 ± 2. μg*/*μL) (*P* = 0.0103), (0.31 ± 0.03 μg*/*μL) (*P* = 0.0139), respectively (Fig. 3A–D). IL-1β levels remained unchanged two weeks after injury (3.06 ± 0.41 μg*/*μL) compared to naïve (2.23 ± 0.25 μg*/*μL) (*P* = 0.1007) (Fig. 3E and F). Levels of IL-6 were under the range of detection in 15 out of 20 samples. However, the in-range samples belonged to SCI animals.

### No histopathologic changes were observed in the lungs after acute SCI

**Figure 4:**
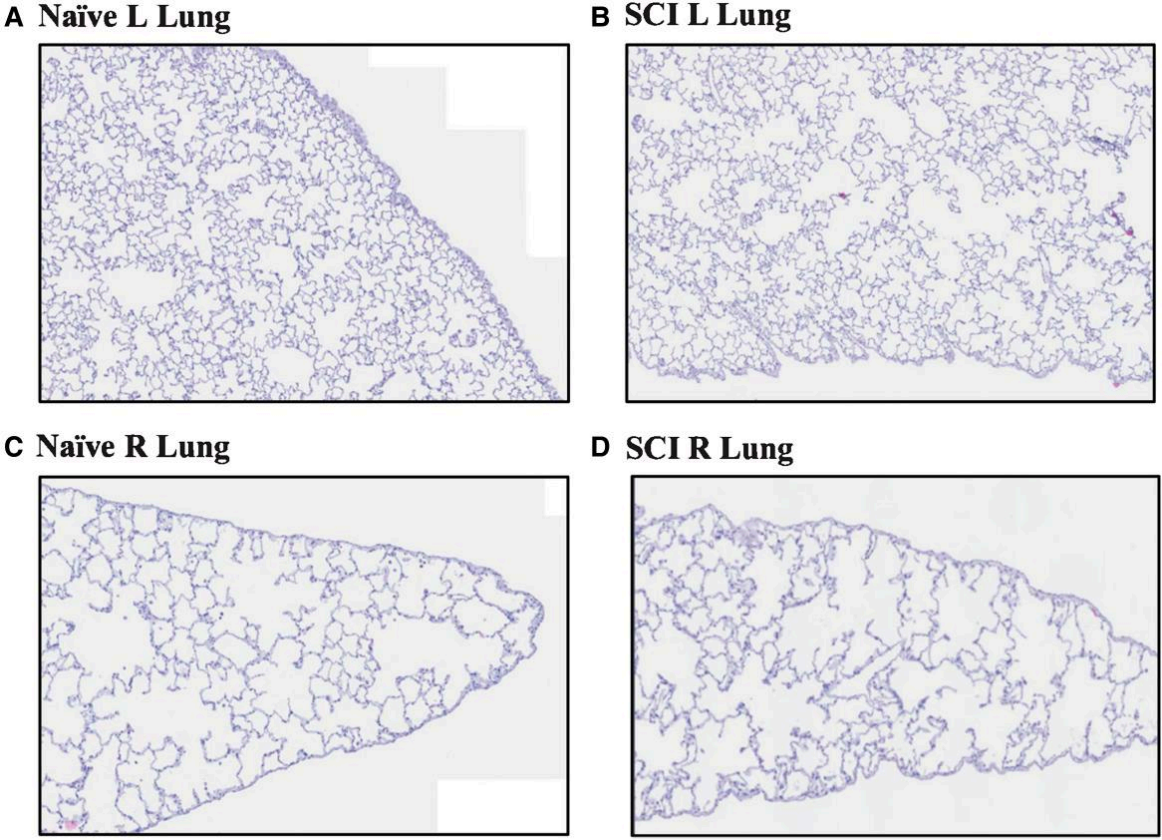
**Histopathology.** No histopathologic differences were observed in the lungs from naïve and two weeks post-SCI rats. Lungs were essentially normal (H&E stain, low power). Representative naïve left lung (**A**; *N* = 5). Representative SCI left lung with scattered parabronchial lymphoid aggregates (**B**; *N* = 5). Representative naïve right lung (C; *N* = 5). Representative SCI right lung (**D**; *N* = 5).

Double-blinded histological analysis was performed by a board-certified clinical pathologist using H&E staining revealed similar findings in both SCI and naïve animals (Fig. 4). More specifically, both groups had rare scattered small foci containing foamy pulmonary alveolar macrophages, mild chronic interstitial inflammation, and mild interstitial fibrosis. However, the majority of the examined lung parenchyma was relatively normal in appearance in all cases. With a more severe ALI, we would expect to see an accumulation of intra-alveolar immune infiltrates, alongside more robust and diffuse interstitial inflammatory infiltrate and microvascular congestion. Lack of histopathology features suggest we are at early stages of lung injury.

### SCI alters lung metabolism

**Figure 5:**
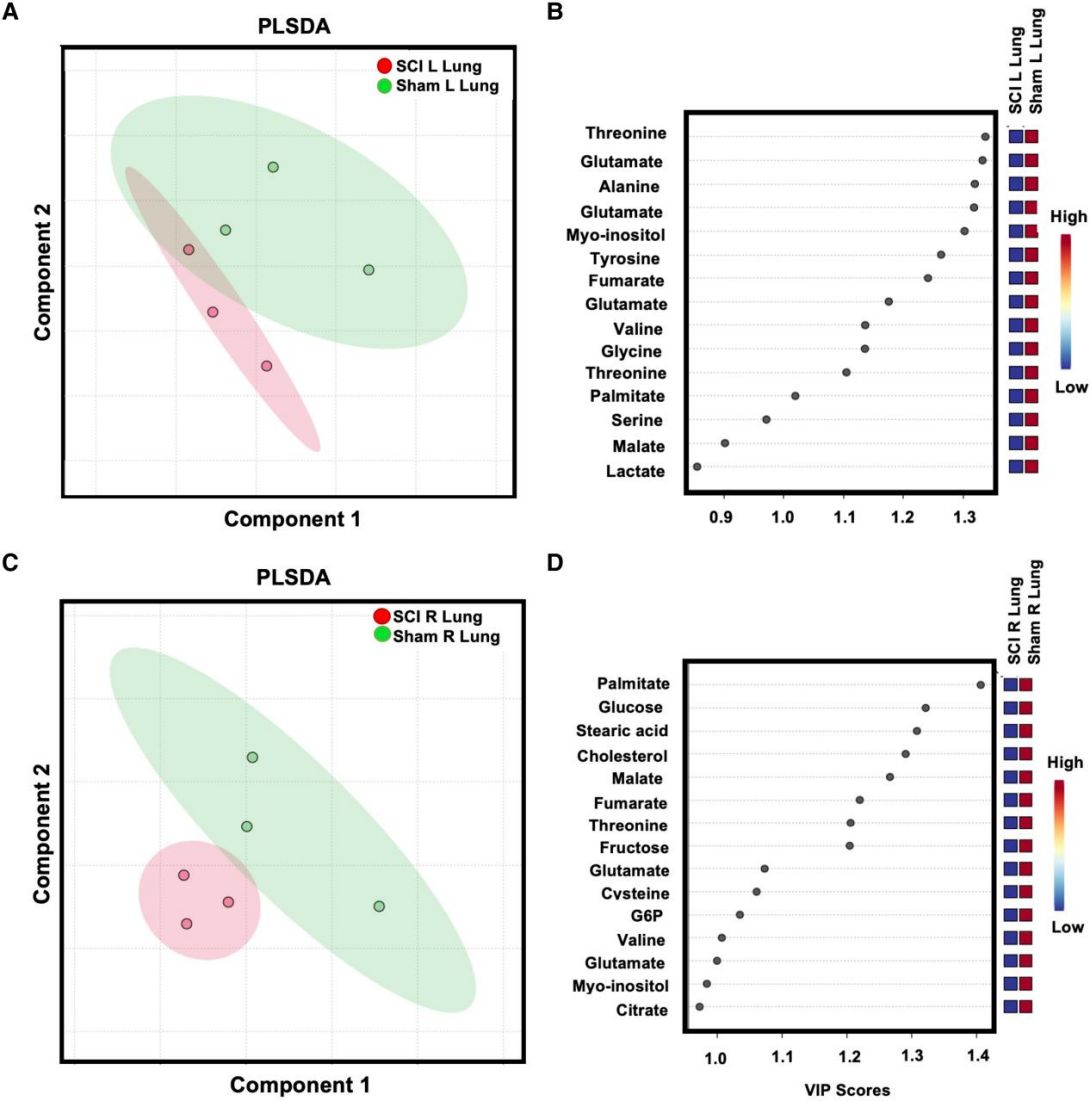
**SCI alters lung metabolites.** Partial least-squares discriminant analysis shows 2D clustering of SCI and sham left lung tissue (**A**). Variable importance in projection (VIP) scores of metabolites from the PLSDA in Panel A (**B**). PLSDA of right lung tissue following SCI (**C**). Variable importance in projection (VIP) scores of metabolites from the PLSDA in Panel C (**D**).

**Figure 6:**
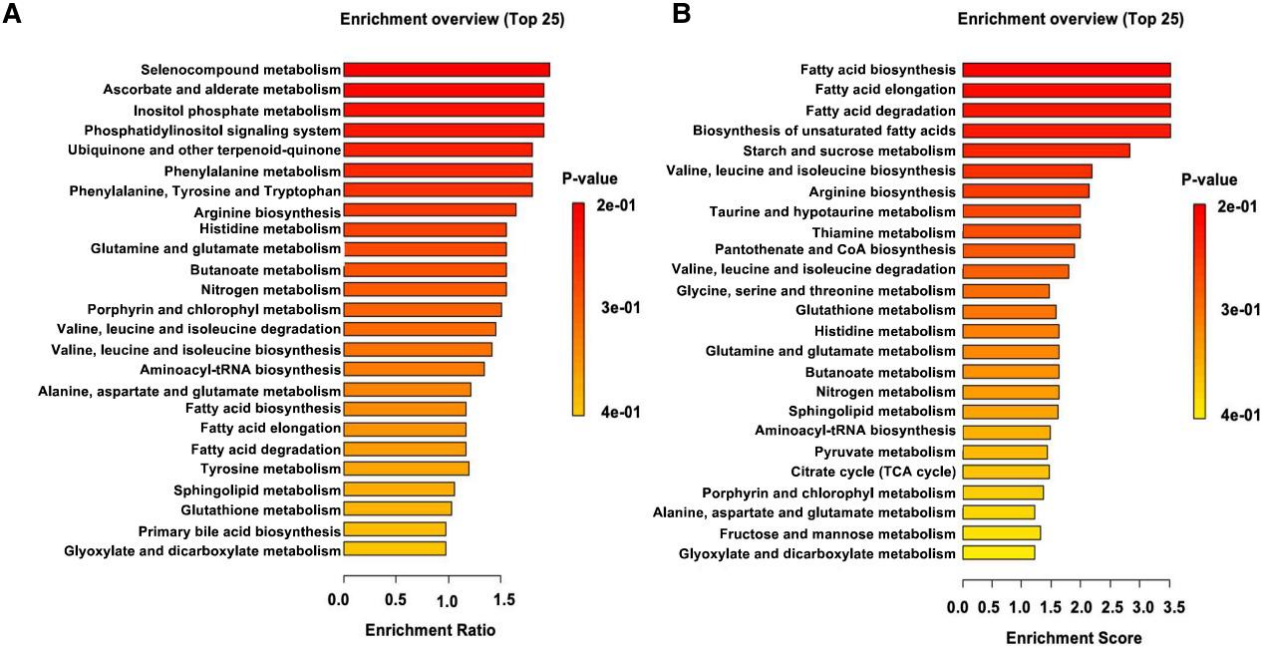
**SCI alters lung metabolic pathways.** Pathway enrichment analysis of all metabolites from left lung tissue (**A**). Pathway enrichment analysis of all metabolites from right lung tissue (**B**). Enrichment ratio is computed by hits/expected hits for each pathway.

Since no obvious histopathologic features were observed, we tested whether lung metabolism was altered after SCI. We performed untargeted metabolomics analysis in both left and right lungs of SCI and sham cohorts of animals. First, we performed multivariate analyses to evaluate differences between sham and SCI rats. Both left and right lungs showed clear separation between SCI and sham cohorts suggesting perturbed metabolism after SCI (Fig. 5). Using variable importance in projection (VIP) analysis, we identified major decreases in pooled metabolites such as amino acids (glutamate, alanine, glutamate, glycine) and lipids, including palmitate and myo-inositol (Fig. 5). Finally, we performed metabolite set enrichment analysis using the metabolomics datasets and observed major perturbed pathways that included many amino acid metabolic pathways and lipid metabolic pathways in agreement with the partial least squares discriminant analysis (PLS-DA) and VIP analysis (Fig. 6). Collectively, these data suggest SCI results in major metabolic reprogramming of the lungs following SCI.

### SCI is associated with changes in N-linked glycosylation of the lung

**Figure 7:**
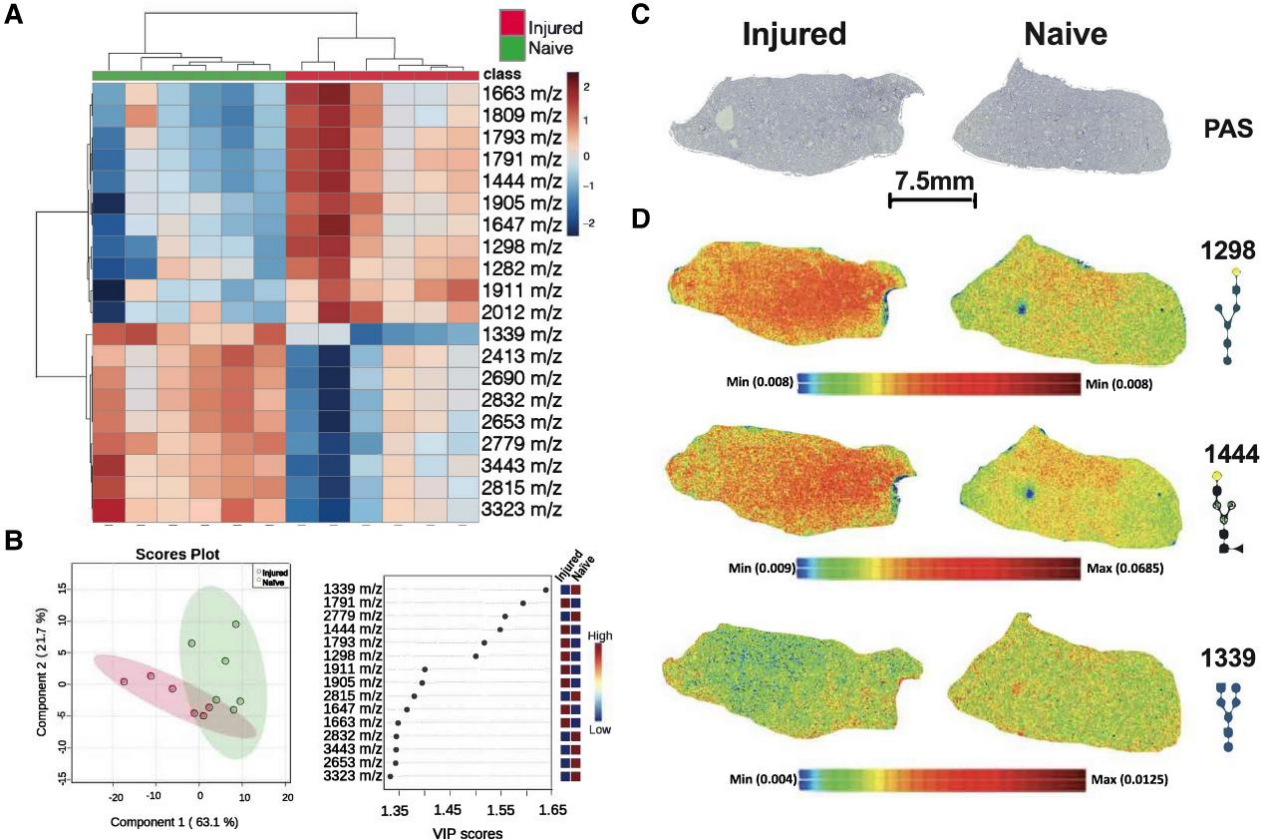
**Changes in N-linked glycosylation in the left lung after SCI.** MALDI mass spectrometry imaging analysis of the left lung following SCI (*N* = 5). Multivariant clustering heat analysis shows separation of SCI and naïve lungs using the top 25 most changed N-linked glycans (**A**). PLS-DA analysis shows 2D clustering of SCI and naïve rat lungs (**B** left panel). VIP scores of most changed N-glycans in whole lung (**B** right panel). The VIP score is calculated as a weighted sum of squared correlations between the PLSDA components and the variable. There was no significant PAS difference between the left lungs from SCI and naïve rats (**C**; *N* = 5). Representative images of selected N-glycans and their spatial distribution across the whole lung (D; *N* = 5).

**Figure 8:**
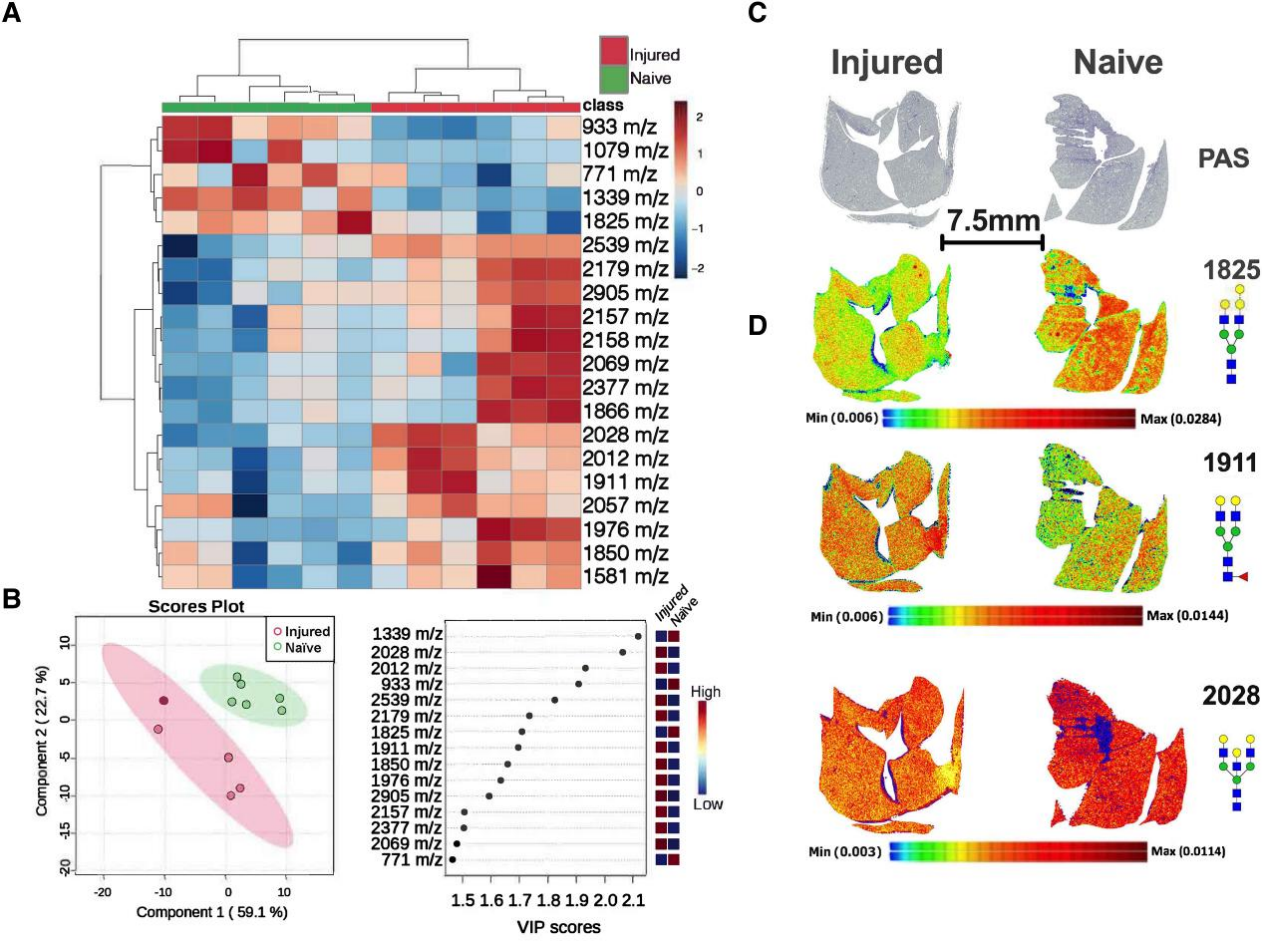
**Changes in N-linked glycosylation in the right lung after SCI.** MALDI mass spectrometry imaging analysis of the right lung following SCI (*N* = 5). Multivariant clustering heat analysis shows separation of SCI and naïve lungs using the top 25 most changed N-linked glycans (**A**). PLS-DA analysis shows 2D clustering of SCI and naïve rat lungs (**B** left panel). VIP scores of most changed N-glycans in whole lung (**B** right panel). There was no significant PAS difference between the right lungs from SCI and naïve rats (**C**; *N* = 5). Representative images of selected N-glycans and their spatial distribution across the whole lung (D; *N* = 5).

In order to further investigate metabolic changes in the lungs caused by SCI, we performed crude carbohydrate analysis of the lung that revealed broad changes in complex carbohydrates (Figs 7A and 8A).^27-29^ However, similarly to H&E analysis, periodic acid-Schiff staining showed no detectable changes between naïve and SCI lungs (Figs 7C and 8C). We next investigated whether N-linked glycan changes occur in ALI by performing *in situ* analysis of N-linked glycomics using enzyme-assisted MALDI-MSI. First, we performed multiple multivariate analysis that included supervised clustering heatmap and PLS-DA on both left and right lungs following SCI (Fig. 7A and B and Fig. 8A and B). Both clustering heatmap and PLS-DA analysis uniquely separated naïve and SCI injured lungs (Fig. 7A and B and Fig. 8A and B). Interestingly, VIP values from PLS-DA revealed heterogenous changes in N-link glycans between left and right lungs (Figs 7B and 8B). Finally, we performed gross tissue structural analysis using the MALDI dataset and found that changes in glycans are consistent across entire lung regions and not confined to specific regions (Figs 7D and 8D). Collectively, MALDI imaging analysis of N-linked glycomics suggests distinct aberrant N-glycan metabolism is associated with ALI after SCI and that these changes are lobe dependent. Further, spatial analysis showing universal changes across entire lobes suggests the possibility of mechanical defects after SCI rather than localized cellular damage.

## Discussion

A majority of individuals with SCI develop secondary complications within the first two weeks of injury.^30^ Specifically, individuals with cervical SCI have a high incidence of respiratory complications and can develop ARDS/ALI after injury.^9,31^ We investigated this clinical observation in the rat model in an effort to capture the effects of SCI on the lungs. Overall, this study demonstrates that mild lung injury and metabolic changes occur following cervical SCI. We observed signs of mild ARDS/ALI at two weeks after cervical SCI with trends towards greater injury in the lung ipsilateral to SCI. These findings establish a model of ALI/ARDS at an acute time point that can be used to characterize and monitor the progression of lung injury after SCI, as well as a means to investigate potential therapeutic strategies.

ARDS/ALI is a progressive disease with various forms of damage occurring at different timepoints depending upon the initiating event. The temporal developments of lung injury following SCI have not been established in the rat model.^32^ However, our data appear to indicate the exudative stage of ALI, with evidence of edema and infiltration of cells into the alveoli without indications of fibrosis. These observations two weeks after SCI suggest a disruption of the epithelial-endothelial barrier in the alveoli.

ARDS/ALI characteristically results in the increased permeability of the alveolar-capillary barrier. The damage of this barrier facilitates the influx of fluid into the alveoli. We assessed edema by calculating the wet/dry ratio for each lung and we found that animals with SCI had increased edematous fluid in both the left and right lungs compared to uninjured controls. Interestingly, despite utilizing a unilateral SCI model, there was no significant difference in edema between the left and the right lung, suggesting that our model of SCI did not specifically target either lung. However, there is a trend that the ipsilateral lung had a greater wet/dry ratio compared to the contralateral lung in SCI animals. Future experiments will include an increased sample size to evaluate the significance of this trend.^22^ Another indicator of lung injury is the recruitment of immune cells into the airspace of the lungs. As expected, we observed primarily alveolar macrophages in the cell counts of uninjured animals. After SCI, the population of cells in the alveoli increased primarily due to the recruitment of neutrophils. Because we observed a substantial increase in both total cell number and neutrophils in the BAL after SCI, we evaluated KC/GRO as it is known to mediate neutrophil recruitment and activation.^33^ We found an increase in KC/GRO in the BAL following SCI. We demonstrated that the increase in neutrophils and KC/GRO was present 14 days following SCI. KC/GRO has functional homology with IL-8 with chemotactic and proinflammatory activity in the rodent.^34^ High levels of KC/GRO in the BAL from ARDS/ALI patients are associated with increased neutrophils in the injured lungs.^35^

Knowing that KC/GRO levels were elevated, we assayed cytokines involved in its inflammatory cascade.^36^ We observed increased levels of TNF-α in the collected BAL fluid from SCI rats. TNF-α has been proposed as a mediator of lung injury and its neutralization ameliorates the injury.^24,25^ Interestingly, there were no significant changes in IL-1β levels after SCI and IL-6 levels were undetectable in naïve and SCI animals. The inability to detect changes in IL-1β and IL-6 may be due to these cytokines’ varying temporal profiles, with IL-6 peaking hours after induction of lung injury in the mouse model, for example.^20^ However, more timepoints need to be evaluated after SCI to provide further insights into the progression of lung injury following SCI.

Increased protein concentration in the BAL can be another indicator of lung injury and vascular permeability in both rodents and humans.^37^ Damage to the alveolar-capillary barrier allows exudate to enter the lungs at the level of the capillaries.^38^ However, despite increased wet/dry ratios in SCI rats, we did not find substantial increases in protein concentration in the BAL after SCI. One potential explanation for this finding is that fluid accumulation may have occurred due to increased hydrostatic pressure rather than loss of the alveolar-capillary barrier function, signaling a transudative effusion. However, we do not have direct evidence for this since we did not measure pulmonary vascular pressures in this model. Our injury model is one of an indirect induction of lung injury, consistent with other forms of trauma.^39^ With the paralysis of the left hemidiaphragm as opposed to the entire diaphragm, the injury is not as severe as a complete transection or high-impact bilateral contusion SCI.

After 14 days, SCI rats showed no histopathologic differences compared to the control rats. The vast majority of the examined lungs were histologically unremarkable with both groups showing rare foci of non-specific findings. However, the presence of these limited findings in both the SCI rats and controls suggests that they are unrelated to the induced SCI. Additionally, the lack of prominent indications of diffuse alveolar damage is consistent with the lack of increased protein in the BAL. These results were expected in the acute setting of SCI, as there has likely not yet been a long enough time-course to result in significant pathologic findings. The changes that we found in Figs 1–3 may manifest into histopathological damage in more chronic timepoints following SCI. Although no obvious histopathological features were detected, we saw perturbed metabolism in the SCI lungs that suggest metabolic reprogramming may predate histopathology. Given that no physical trauma was caused to the lung during SCI, we speculate that disrupted signaling events between the central nervous system and the lung following SCI caused early metabolic disruptions that would lead to chronic lung injury. Future experiments should include analyses of chronic time points post SCI and associate metabolic changes with acquired histopathology.

We investigated changes in N-linked glycan metabolism as a potential early indicator of the development of lung injury. N-linked glycan biosynthesis is an understudied facet of glucose metabolism. In the lung, N-linked glycans are crucial for the differentiation of bronchoalveolar stem and alveolar type 2 cells to form the alveolar and bronchiolar lining.^40-42^ Further, N-linked glycans are critical components of mucins and surfactant proteins that maintain the air–liquid interface, reduce surface tension, and provide lubrication for the mechanical action of the lung.^43,44^ Collectively, MALDI imaging analysis of N-linked glycomics suggests distinct aberrant N-glycan metabolism is associated with ALI after SCI and that these changes are lobe dependent. Further, spatial analysis shows universal changes across the entire left and right lobes. These findings support the possibility of widespread changes in metabolism after SCI that could potentially impact lung progenitor cell differentiation, oxygen/CO_2_ exchange, and movement of the lung. With these important implications in mind, aberrant N-linked glycan phenotype in our ALI model warrant further investigation.

ALI/ARDS is often induced in animal models with a direct inflammatory insult to the lungs.^45^ However, ALI/ARDS can also manifest as a result of indirect insults such as sepsis, burns, and trauma via inflammatory mechanisms.^39,46^ After SCI and other forms of neurotrauma, there is an intense systemic inflammatory response that affects whole organ systems.^47,48^

After neurotrauma, circulating proteins serve to activate the inflammasome in type II alveolar epithelial cells of the lungs. This level of activation of the inflammasome and the resultant lung injury is not observed in sham and naïve animals—where no difference between the two was found.^49^ Importantly, this demonstrates that the extent of injury and damage in a sham neurotraumatic model had no significant effect on the lungs. Sham animals were not initially included in our study as the injury mimics a vertebral column fracture without an SCI, which has been shown to lead to ARDS in an observational clinical study.^9,49^ We used both sham and naïve animals in the metabolomic analysis to confirm that aberrant metabolic changes occur after SCI and that these changes are due to the injury model itself.

Lung damage has been found in rodent models after thoracic-level SCIs with evidence for systemic inflammation as the cause.^13,50^ However, lung injury after cervical SCI cannot be solely attributed to inflammation. Individuals with cervical SCIs are at higher risk for the development of ARDS/ALI and have more severe ARDS/ALI than at any other injury level.^9^ This increased susceptibility and severity are likely because an injury to the cervical level of the spinal cord affects breathing function while simultaneously generating a general systemic inflammatory response.^51^ Our results suggest this explanation may be the case, as we demonstrate that the lung ipsilateral to the SCI trends towards more severe signs of ARDS/ALI. However, the significance of these differences was not detected potentially due to small sample size.^22^ This observation may be a result of the left C2Hx injury model used in this study. In this model, the innervating signal to the left hemidiaphragm is severed above the level of the phrenic motor nucleus. This injury results in paralysis of the left hemidiaphragm, leaving the operation of the right hemidiaphragm intact. The desuetude of the left hemidiaphragm affects the pressure-differential in the left pleural cavity and lowers the tidal volume of injured animals.^52^ With a reduced capacity of the lungs to perform gas-exchange, ALI/ARDS can ensue.^53^ Future experiments will be directed toward elucidating the side-specific differences in the lungs following unilateral SCI. These studies will include direct comparisons between SCI and sham injured animals.

We provide a means to investigate potential therapeutic strategies to ameliorate the onset and progression of lung injury after SCI in the rat model. Based on our collective findings, it would be pertinent to look to the aberrant metabolic profile of the lungs following SCI. Specifically, advances are being made in glycan-based immunotherapeutics in the treatment of breast and lung cancer.^54^ A broader use of these therapeutics may one day be applied to lung injury after SCI to target the changes in metabolism we observe in the lungs following SCI.

In summary, our studies demonstrate that animals with a cervical SCI display signs of mild ALI/ARDS including, but not limited to marked increases in alveolar neutrophil counts, heightened levels of proinflammatory cytokines and neutrophil-activating KC/GRO, intra-alveolar edema, and metabolic dysregulation 14 days after injury. Collectively, this study establishes an SCI-induced ALI model that can be developed further and used to identify potential targets to ameliorate respiratory distress and lung injury after SCI.

## Abbreviations

ALI =: acute lung injury
ARDS =: acute respiratory distress syndrome
BAL =: bronchoalveolar lavage
BCA =: bicinchoninic acid
C2Hx =: hemisection at the second level of the cervical spinal cord
DPI =: days post injury
EDTA =: ethylenediaminetetraacetic acid
FFPE =: formalin fixed paraffin-embedded
GCMS =: gas chromatography mass spectrometry
H&E =: hematoxylin and eosin
IL-1B =: Interleukin 1 beta
IL-4 =: Interleukin 4
IL-6 =: Interleukin 6
KC/GRO =: keratinocyte chemoattractant and human growth-related oncogene
MALDI-MSI =: matrix-assisted laser desorption/ionization mass spectrometry imaging
MSD =: meso scale discovery
PAS =: periodic acid-Schiff stain
PLS-DA =: partial least squares discriminant analysis
SCI =: spinal cord injury
TNF-α =: tumor necrosis factor alpha
VIP =: variable importance projection
